# Elicitation from virus-naive individuals of cytotoxic T lymphocytes directed against conserved HIV-1 epitopes

**DOI:** 10.1186/1476-9433-5-1

**Published:** 2006-05-18

**Authors:** Pedro A Reche, Derin B Keskin, Rebecca E Hussey, Petronela Ancuta, Dana Gabuzda, Ellis L Reinherz

**Affiliations:** 1 Laboratory of Immunobiology and Department of Medical Oncology, Dana-Farber Cancer Institute, 44 Binney Street, Boston, MA 02115, USA; 2 Harvard Medical School, Boston, MA 02115, USA; 3 Department of Cancer Immunology and AIDS, Dana-Farber Cancer Institute, 44 Binney Street, Boston, MA 02115, USA

## Abstract

Cytotoxic T lymphocytes (CTL) protect against viruses including HIV-1. To avoid viral escape mutants that thwart immunity, we chose 25 CTL epitopes defined in the context of natural infection with functional and/or structural constraints that maintain sequence conservation. By combining HLA binding predictions with knowledge concerning HLA allele frequencies, a metric estimating population protection coverage (PPC) was computed and epitope pools assembled. Strikingly, only a minority of immunocompetent HIV-1 infected individuals responds to pools with PPC >95%. In contrast, virus-naive individuals uniformly expand IFNγ producing cells and mount anti-HIV-1 cytolytic activity. This disparity suggests a vaccine design paradigm shift from infected to normal subjects.

## Background

Although it has been more than 20 years since the discovery that HIV-1 is the cause of acquired immune deficiency syndrome (AIDS), we are not yet close to realizing a vaccine to halt the devastation created by the AIDS pandemic [1]. HIV-1 clearance by the human host immune system and development of effective natural immunity have never been observed in AIDS patients. Consequently, correlates of immune protection are unknown (reviewed in [2]). Several features of HIV-1 biology have resulted in unprecedented challenges to vaccine development [3,4]. First, the virus has extraordinary genetic diversity on a global population scale as well as at the level of the single infected person [5,6]. With up to a billion new viral particles produced per day in an infected individual, HIV-1 genetic variability can be greater within one host than the worldwide variability of influenza A virus in any year. This sequence/epitope variability thwarts effective cellular and humoral immune responses. Second, HIV-1 infection targets CD4^+ ^helper T cells, thereby blunting mechanisms that normally amplify adaptive immunity. Third, HIV-1 is difficult to neutralize because the viral envelope glycoproteins are protected by a glycan shield, and exist in several distinct conformations, rendering conserved epitopes largely inaccessible to antibody-mediated neutralization [4].

CD8^+ ^cytotoxic T lymphocytes have been shown to contribute to HIV-1 containment through direct killing of infected cells and production of soluble anti-viral mediators [7-9]. In one study, AIDS patients with high anti-HIV-1 specific CD8^+ ^CTL levels in blood generally exhibit better clinical status [10]. Furthermore, *in vivo *containment of HIV-1 replication appears coincident with the emergence of specific CTL, well before neutralizing antibody [8]. Conversely, viral mutations resulting in loss of HIV-1 or simian immunodeficiency virus (SIV) recognition by CTL are linked to increased viral replication *in vivo *and diminished immune function [11,12]. Experimental depletion of CD8^+ ^T cells prior to SIV infection in monkeys leads to uncontrolled viral replication, accelerated disease and death [13]. Collectively, these data suggest that the induction of CTL responses to an appropriate selection of viral CTL epitopes prior to HIV-1 exposure might comprise one important element in HIV-1 vaccine development. This current study addresses elicitation of CTL against conserved HIV-1 epitopes using blood from healthy donors and the implications of our findings on future preventive HIV-1 vaccine designs.

## Methods

### Generation of a consensus HIV-1 proteome

A consensus HIV-1 proteome bearing a consensus sequence of the HIV-1 gene products (GAG, POL, ENV, VIF, TAT, REV, VPU/VPX, VPR, and NEF) with the variable residues masked was obtained as indicated below. First, multiple sequence alignments for the relevant HIV-1 gene products including representative sequences from all HIV-1 clades were retrieved from the HIV databases in Los Alamos [14] (Table 1). Then, sequence variability in the alignments was computed using a variability metric (V) formally identical to the Shannon entropy equation [15]. Briefly, V per site is given by:

**Table 1:** HIV-1 protein sequences used in this study

	Sequences*																		
GENE	Total	A-A1	B	C	D	F1	F2	G	H	K	O	01	02	04	06	10	11	12	CPZ

GAG	207	10	37	28	5	7	2	4	3	3	5	14	8	3	4	3	4	3	5
POL	204	11	34	26	5	4	3	4	3	2	5	14	12	3	4	3	5	3	5
ENV	408	24	128	51	17	5	5	9	3	2	10	33	16	3	5	3	6	3	5
VIF	427	28	190	40	13	5	5	5	3	2	24	15	12	3	4	3	4	12	5
TAT	219	11	39	29	7	6	3	4	3	2	4	14	14	3	6	3	4	3	5
REV	231	13	39	38	10	6	3	4	3	2	4	17	12	3	4	3	4	3	5
VPU/VPX	333	14	87	50	18	6	3	6	3	2	15	25	14	3	4	3	4	3	5
VPR	368	9	142	44	7	4	3	5	3	2	25	16	12	3	4	3	4	3	5
NEF	449	12	282	44	4	4	3	7	5	2	4	31	12	3	4	3	11	3	4

*HIV-1 sequences were obtained from the HIV database for the indicated clade categories.



where P*i *is the fraction of residues of amino acid type *i*, and *M *is equal to 20, the number of amino acid types. V ranges from 0 (total conservation, only one amino acid type is present at that position) to 4.322 (all 20 amino acids are equally represented in that position). Note that in order to achieve the maximum value *V *= 4.3, at least 20 sequences are required. Gap symbols (-) were considered for deriving the consensus sequence but are not computed for the variability calculations. Finally, consensus sequences with the variable positions masked were obtained as follows. A variability threshold (*Vt*) was set 1.0, and at each position in the alignment *V *was compared to the *Vt*, if *V *≤ *Vt *the most frequent residue is selected as the consensus, otherwise if *V *> *Vt *a "." symbol is used to mask the sequence at that position (the HIV-1 consensus proteome generated in such a way is shown in Additional file 1).

### Prediction of peptide-HLA I binding and determination of the HLA I-binding profile of CTL epitopes

Peptide-MHCI binding predictions were obtained using position-specific scoring matrices (PSSMs), also known as weight matrices or profiles, which are obtained from aligned peptides known to bind to the relevant MHCI molecules [16]. PSSMs used in this study were all obtained from 9mer peptides, and thereby predicted peptide binders were all 9mers. Using this approach it is been shown that ≥ 85 % of true peptide-MHCI binders are found among the top 2% scoring peptides of a given protein [16]. The potential binding of each selected CTL epitope to 55 HLA I molecules for which representative PSSMs are available was evaluated as follows: A) the epitope sequence is appended to a random protein of 1000 amino acids in length (always the same) with an amino acid distribution obtained from HIV-1 sequences; B) peptide-MHCI binding predictions from this random protein are obtained using the corresponding PSSMs; C) binding of the CTL epitope to an HLA I molecule is considered to occur if its sequence is found among the top 2% ranking peptides. Cycling through the above steps results in a table consisting of a predicted HLA I binding profile for each CTL epitope.

### Computing the cumulative phenotype frequency

Given a set of HLA I alleles (N), their cumulative phenotypic frequency (CPF) in a given population can be computed from the gene and haplotype frequencies of the individual alleles using the equation 2.



where the EPF_i _term account for the Expected Phenotypic Frequency of each of the alleles (i), and the ECPF*ij *term accounts for the Expected Combined Phenotypic Frequency of each two distinct pair of alleles (*ij*) in the *N *set. EPFi is calculated through equation 3:

EPFi = 2xGFi - (GF_i_)^2 ^    Eq. 3

where GF_i _is the gene frequency of the allele *i*. On the other hand, given a pair of alleles (*ij*), ECPF_ij _is computed upon considering whether they belong to the same HLA I gene (HLA-A, -B, -C) or not. If they do belong to the same gene, we assume a Hardy-Weinberg equilibrium and calculate ECPF *ij *as follows:

ECPF_ij _= 2xGF_*i *_xGF_*j*_;     Eq. 4

However, if the two alleles belong to different genes and their haplotype frequency is available (HF_*ij*_) we calculate ECPF_ij _using the following formula:

ECPF_ij _= 2xHF_*ij *_- (HF_*ij*_)^2 ^    Eq. 5

If no haplotype frequency is available, ECPF_ij _is computed as if they belonged to the same gene using equation 4. In this 'study, HLA I allelic and haplotype frequencies used for the above calculations were those published by Cao et al [17], which corresponded to 5 major American ethnic groups (Black, Caucasian, Hispanic, Native American, and Asian). Note that for computing CPF, we have used a method similar to that of Dawson et al. [18] which, unlike the methods of Schipper [19] and Gulukota [20], considers haplotype frequencies and hence linkage disequilibrium. However, in contrast to Dawson et al., we minimized the number of peptides to be included in the vaccine rather than the alleles that are required to be the binding targets for those peptides.

### Selection of minimal a set of epitopes providing a determined population protection coverage (PPC)

For a set of CTL epitopes, the PPC defines the population proportion that could potentially mount an immune response to any of these epitopes. Given the required HLA I restriction of CTL responses, the PPC for a set of CTL epitopes matches the proportion of the population that exhibits at least one of the HLA I alleles targeted for binding any of those epitopes, and is in turn given by the CPF of the targeted HLA I alleles. Provided a set of CTL epitopes (N_CTL_) and their binding profiles (HLA I alleles they bind to), an algorithm for the selection of a minimal subset of epitopes (K) providing a PPC ≥ 95% was implemented as follows:

1. We begin by considering all independent subsets (X) of epitopes in N_CTL _of size K (number of epitopes), begining with 2.

2. For each independent subset of epitopes, their HLA I binding profiles are pooled into a single non-redundant array of HLA I alleles, and the CPF is computed for 5 major American ethnicities using the HLA I gene and haplotype frequencies reported by Cao et al [17]. Only epitope subsets reaching a CPF ≥ 95% for the five ethnicities are saved and returned by the algorithm.

3. If none of the independent epitope subsets X of size K is found to reach a CPF ≥ 95% the algorithm increases the subset size by 1 and loops over 1 through 3.

4. The algorithm stops cycling and returns the results when at least 4 independent subsets of epitopes of size K reach a CPF ≥ 95%. If only 3 independent sets of size K are found to reach a CPF ≥ 95%, the program will cycle once more and find the subsets of size K + 1 that reach a CPF ≥ 95%, and then exit.

### HIV-1 specific CTL epitopes and assembly of peptide pools

The 25 peptides considered in this study consisting of HIV-1 specific CTL epitopes were synthesized by Invitrogen Co. (Carlsbad, CA). HPLC analysis showed that the purity of the synthesized crude peptides was 86–92%. All peptides had expected masses as confirmed by mass spectrometry. Crude peptides were used for initial peptide binding assays and selected crude peptides were purified to >96% by reverse phase HPLC for further experiments. Several peptide pools were obtained by combining an excess number of CTL epitopes providing a PPC ≥ 95%, with peptide pool #1 containing all selected peptides and smaller pools #2–#5 as defined in Results. All peptides pools were prepared in DMSO at 200 μM each.

### Generation of HIV-1 specific T cell lines

Langerhans-like APC cells were differentiated from adherent donor monocytes in DMEM medium supplemented with 20% donor serum, L-glutamine (2 mM), penicillin (100 U/ml), streptomycin (100 μg/ml), 2-ME (50 μM). (DMEM complete), GM-CSF (50 ng/ml), IL-4 (50 ng/ml) and TGFβ 1 (10 ng/ml)(Peprotech) for one week. Differentiated Langerhans cells were matured with 1 μg/ml Peptidoglycan (Sigma) overnight. Cells were pulsed with HIV-1 peptide pools (10 μM total) for 3 h and irradiated (3000 rad). Fresh donor PBMC were prepared by Ficoll-Paque (Amersham Biosciences) centrifugation. Donor PBMC were plated at a density of 10^7^cells/ml together with 5 × 10^4^/ml peptide loaded irradiated donor Langerhans cells in 24 well culture plates in complete DMEM medium with 10 μM HIV-1 peptide pools. Cultures were fed with 10 u/ml IL-2 (BD biosciences) five days after stimulation and re-stimulated with peptide loaded irradiated (3000 rads) fresh donor PMBC every week. Replicate 96 well plates were pulsed with 1 μCi per well ^3^H Thymidine after 4 days of culture, harvested after 18 h, and ^3^H Thymidine incorporation was detected using a β Counter (Perkin Elmer 1450 LSC).

### CTL analysis

A0201^+ ^TAP- deficient T2 hybridoma target cells (ATCC) or A2 supertype transfected 721.221 cell lines were plated at a density of 10^6 ^cells/ml in 24 well plates. Cells were pulsed with 10 μM A0201-restricted HIV-1 peptide pool or 10 μM A0201restricted HTLV-TAX as negative control peptide for 18 hours at 37°C. Cells were washed twice with DMEM media (20% fetal calf serum, L-glutamine (2 mM), penicillin (100 U/ml), streptomycin (100 μg/ml), 2-ME (50 μM). (DMEM media). and pulsed with 100 μC of ^51^Cr for 90 minutes at 37°C. Target cells were washed three times with serum free OptiMEM media (Gibco) to remove excess ^51^Cr and plated with sorted HIV-1 specific CD8^+ ^T cells at 30:1, 10:1, 3:1 and 1:1 ratios. After four hours of incubation 50 μl of culture supernatant were mixed with liquid scintillation cocktail (Perkin Elmer Optiphase supermix) and analyzed for ^51^Cr release using a Luminescence Counter (Perkin Elmer 1450 LSC). Percent specific chromium release was calculated using the formula [(Experimental release- spontaneous release)/(maximum release in 5% Triton-x100- spontaneous release)]. To analyze CTL responses to naturally processed HIV-1 epitopes, TAP sufficient T1 hybridoma target cells T1 (A0201+, B5101^+^, CD4^+^) were infected with 0.5 MOI HIV-IIIB per cell for 3 hours. Cells were washed and plated at a density of 10^6^/ml in DMEM media. 2–3 days later, HIV-IIIB infected T1 cells were used in ^51^Cr release assay as described above. Intracellular p24 staining by FACS was used to demonstrate that essentially all target cells were HIV-IIIB-infected.

### IFNγ-ELISPOT assay on HIV-1 infected donor cells

CD8^+ ^T-cell responses to pools of HIV-1 epitopes were quantified by gamma interferon (IFN-γ) ELISpot assay as follows. Peripheral blood mononuclear cells (PBMC) isolated from HIV-1 infected patients were plated at 100,000 per well with peptide pools at a final concentration of 10 μM in anti-interferon gamma mAb 1-D1K (Mabtech, Stockholm, Sweden) coated polyvinylidene 96-well plates (Millipore.MA) and processed as previously described [21]. For each individual peptide, the assay was run in triplicate. Negative and positive controls were obtained by incubating individual PBMC with medium alone (negative control) and phytohemagglutinin (PHA) as a positive control for naive patients. For the assessment of general immunocompetence of HIV-1 infected patients, a CEF peptide pool was used as an internal positive control. The CEF pool consists of optimal T cell epitopes for CMV/EBV and Influenza viruses provided by the NIH reagent program. Only HIV-1 patients whose blood yielded positive CEF and/or PHA responses were chosen as subjects for the present study.

The number of specific IFN-γ secreting T cells was determined with an automated ELISPOT reader (AID, Strassberg, Germany), calculated by subtracting the average negative control value and expressed as the number of spot-forming cells (SFC) per 10^6 ^input cells. Negative controls were approximately 50 SFC per 10^6 ^input cells for HIV-1 infected patients and around 40 for naïve subjects. A response was considered positive if there were 50 SFC per 10^6 ^input cells and the activity was at least three times as great as the mean background activity.

### IFNγ – production on HIV-1 specific T cell lines generated from naive donors

HIV-1 peptide pool-specific T cell lines were generated as described above. Two weeks after the second stimulation, 25,000 per well HIV-1 specific T cells were plated into anti-interferon gamma mAB 1-D1K (Mabtech) coated polyvinylidene 96-well plates (Millipore) with 25,000 per well irradiated donor PBMCs and stimulated with 10 μM of each HIV-1 peptide pool. Plates were processed and spot forming cells were calculated as described above.

### HLA-A 0201-binding assay

A0201^+ ^TAP- deficient T2 hybridoma cells (ATCC) were plated at a density of 10^6 ^cells/ml in 24 well plates. Cells were pulsed with 10 μM A0201 restricted HIV-1B peptides, 10 μM A0201restricted HTLV-TAX (LLFGYPVYV) positive control peptide and 5 μg/ml β 2 microglobulin (BD biosciences) for 18 hours at 37°C in serum free AIM5 media (Gibco). A0201 expression was determined by flow cytometry (FACSAria) using FITC-conjugated BB7.2 mAb (BDbiosciences). Mean cell fluorescence (MCF) intensities were normalized to HTLV-TAX positive control peptide using the formula [(MCF.sample- MCF.control)/(MCF.HTLV-TAT-MCF.control)].

### DimerX staining

A2:Ig fusion protein DimerX (BD Biosciences) was passive loaded with individual A0201 restricted HIV-1B peptides or positive control EBV peptide at 640 molar excess in PBS pH 7.2 overnight. HIV-1B specific CD8^+ ^cells were stained with peptide loaded A2:Ig fusion protein DimerX reagent and detected by PE-conjugated A85-1 mAb (anti-mouse IgG1, BD biosciences). DimerX staining was determined by flow cytometry (FACSAria, BD Biosciences). The negative control straining was obtained using the HIV-1 specific T cell line and a non-relevant HIV-1 peptide loaded DimerX. The positive control was obtained using donor PBMC's stimulated with 1uM BMLF-1 EBV peptide for one week and then stained with EBV peptide loaded Dimer X.

### Generation of HLA allele specific transfectants for peptide binding and restriction analysis

#### Cell lines and RNA isolation

HLA-homozygous human B-lymphoblastoid cell lines (B-LCL) from the International Histocompatibility Working Group (IHWG) were thawed and grown in RPMI complete medium (RPMI supplemented with 10% FCS, 2 mM L-glutamine, 1 mM sodium pyruvate, and 1% pen-strep). The HLA class I deficient 721.221 LCL line and the Amphopack 293, retroviral producer line, were maintained in DMEM complete medium (DMEM supplemented with 10% FCS, 2 mM L-glutamine, 1% pen-strep, 1 mM sodium pyruvate). Total RNA was isolated from 5 × 10^6 ^B-LCL using the RNeasy kit (Qiagen) according to the manufacturer's instructions. The purified RNA was eluted in 50 ul RNase-free water and 10 μl of this was used to synthesize cDNA using an oligo dT primer, dNTP mix, DTT, and M-MLV reverse transcriptase (200 u) (Gibco BRL).

#### Primer, PCR amplification and cloning of PCR products

HLA locus-specific primers were synthesized according to the information from [22]. Sense and antisense primers were made for each of HLA-A, HLA-B, and HLA-C with a HindIII site at the 5' end of sense and a NotI site at the 5' end of the antisense. PCR amplification was done using 2 μl of cDNA, 1× PCR buffer with Mg, 200 uM each dNTP, 0.3 uM of each primer and 0.5 μl Taq polymerase. The conditions for PCR were one cycle of 95°C for 5 min prior to addition of polymerase, followed by 30 cycles of 94°C for 1 min, 58°C for 1 min, 68°C for 1.5 min and finally one cycle of 68°C for 4 min. PCR products were analyzed on a 1.2 % agarose gel and gel purified using a gel extraction kit (Qiagen # 28704). Subsequently, 6 μl of purified PCR product was ligated into the pCR2.1 vector using the TA cloning kit (Invitogen, K2000-01) following the manufacturer's directions. Transformation into INVaF' competent cells was carried out and plated onto LB/ampicillin (100 μg/ml) plates. After incubation at 37°C overnight, colonies were selected, grown and miniprep DNA prepared (QIAprep Spin Miniprep Kit (Qiagen 27104). Clones were checked for inserts by restriction digestion with EcoR1. Several clones for each were sent for DNA sequencing using the vector primers M13 Reverse and T7 promoter. Two additional primers designed to anneal in the middle of all HLA class I genes in the sense and anitsense directions were also used [22]. All sequences were verified by DNA sequencing.

#### Subcloning of HLA cDNAs into retroviral vector pLNCX2 and generation of viral supernatants

Sequence-verified HLA cDNA clones were digested with HindIII and Not I and the insert was gel purified and subcloned into the retroviral vector, pLNCX2 (BD Biosciences, cat# 6102-1) which had been linearized with HindIII and NotI. Plasmid DNA preps were done and sequence was confirmed once again using the forward and reverse sequencing primers (BD Bioscience #K1060-F) for the vector, pLNCX2. Retroviral producer cell lines were established by transfection of the Amphopack 293 cell line using the HLA/pLNCX2 DNA. Amphopack 293 cells (4 × 10^6^) were seeded onto a 100 mm tissue culture dish in 15 ml of DMEM complete medium and incubated overnight at 37°C. Five minutes prior to transfection, the medium was replaced with complete medium containing chloroquine (25 μM). The semi-confluent cells were transfected using 20 μg of plasmid DNA in combination with 61 μ l2 M CaCl_2 _(0.5 ml total volume) and 0.5 ml 2 × HBS, bubbled in. The solution was added dropwise to the cells and gently swirled. Plates were incubated at 37°C for 6–8 h, at which time the medium was removed and replaced with fresh DMEM complete medium. Plates were incubated further at 37°C for 48 h. At 48 h post-transfection the viral supernatant was removed from the plates and spun at 2000 rpm for 10 min, then filtered through a 0.45 um filter. The supernatant was stored at 4°C for immediate infection or frozen at -80°C for later infection.

#### Retroviral infection of 721.221 cells, flow cytometric analysis and cell sorting

721.221 cells (EBV transfected B cell line which is MHC class 1 A, B, C negative) were infected with the retroviral supernatant using Lipofectamine reagent (Invitrogen #18324-111). For each infection, 1–3 × 10^6 ^cells in log phase were pelleted, resupended in 100 μl of DMEM complete medium, and transferred to one well in a 24 well plate. 4 μl of lipofectamine reagent, 2 ml of viral stock, and 0.2 ml of l M Hepes were added. The plate was spun for 1–2 h at 2000 rpm and subsequently the medium was discarded, the cells were washed one time with fresh medium, and finally were resuspended in 2 ml of fresh medium. The following day, infected cells were put into neomycin (G418) selection at 800 μg/ml. Selection was continued for 5–7 days at which point the concentration was dropped down to 400 μg/ml. Approximately one week post-infection, the cells were analyzed for cell-surface expression of HLA. An aliquot of cells was stained using the monoclonal antibody W6/32 at 1:500, incubated at 4°C for 20 min, washed once (using 1 × PBS containing 2% human AB serum and 1% pen-strep) and then incubated with a second step of Goat-anti-mouse IgG FITC at 1:200 for 20 min at 4°C. The cells were washed twice and then analyzed on the FACS machine. Infected 721.221 cells that showed HLA surface expression (varying from 5% to 60%) were subsequently expanded, stained and sorted for W6/32 positive cells. Sorted cells were expanded in DMEM complete containing 400 μg/ml G418, and aliquots were frozen. The following 721.221 cell lines are available: A0101, A0201, A0202, A0203, A0204, A0205, A0206, A0207, A0301, A1101, A2301, A2402, A2403, A2902, A3101, A3301, A6601, A6801, A6802, B0702, B0801, B1501, B1502, B1510, B2702, B2703, B2705. B2706, B3501, B3801, B3909, B39011, B4002, B4402, B4403, B5101, B5301, B5401, B5502, B5701, C0102, C0304, C0702.

## Results and discussion

### Strategy for selection of HIV-1 specific CTL cell epitopes and generation of peptide pools

In this study, we approached the formulation of CTL targets from a collection of 199 unique HIV-1 CTL epitopes retrieved from the Los Alamos HIV database [14]. The peptides were all 9mers, which is the optimal peptide length for MHCI binding, and are known to elicit CTL responses in one or more HIV-1-infected individuals, indicating that they are naturally processed during the course of infection. Identification of processed epitopes eliminates one hurdle in CTL epitope-based vaccine design. Nonetheless, a successful CTL-based strategy must confront the sequence variability of HIV-1 strains and polymorphism of the human MHCI molecules (HLA I). HIV-1 sequence variation largely contributes to immune evasion. In fact, it has been suggested that at a population level, HIV-1 variants have evolved to adapt to CTL responses [23,24]. Certainly, CTL escape variants can arise from mutations within and/or flanking HIV-1 CTL epitopes [12].

To select target epitopes less likely to escape from CTL, we first subjected representative viruses from all HIV-1 clades (Table 1) to a Shannon Entropy (H) variability analysis [25], thereby removing or "masking out" any residue with H >1. See Additional file 1 for the resulting HIV-1 consensus proteome. All residues in the selected CTL epitopes are therefore quasi-invariant. The most proximal residue flanking the C-terminus of the CTL epitope is a determinant for cleavage by the proteasome, and mutations in that residue can abrogate T cell recognition [26]. Accordingly, we also excluded those CTL epitopes with a C-terminal flanking residue with H >1. As a result, only 37 of the 199 HIV-1 CTL peptides were chosen.

HLA I polymorphisms complicate development of a broadly protective CTL epitope vaccine by limiting population coverage. For a given selection of CTL epitopes, the population protection coverage (PPC) is given by the proportion of cumulative phenotypic frequency (CPF) of the HLA alleles restricting these epitopes. CPF can be computed from the HLA gene and haplotype frequency in the population. Using the HLA I gene and haplotype frequency reported by Cao et al. [17] for five major American ethnicities (Black, Caucasian, Hispanic, Native American, and Asian), we developed an algorithm that first computed the binding of each epitope to 55 HLA I alleles and then selected epitope combinations providing a PPC ≥ 95% for all ethnic groups considered (details in supporting methods). From this analysis, we predicted that as few as five epitopes from the 37 conserved HIV-1 specific CTL epitopes should be recognized by = 95% of the population, regardless of ethnicity. Furthermore, we identified 5-epitope combinations using only 25 of the 37 conserved HIV-1 specific CTL epitopes (Table 2). These 25 CTL epitopes were distributed as follows: POL (reverse transcriptase, integrase and protease), 14 epitopes; GAG, 5 epitopes; ENV, 3 epitopes; and NEF, 3 epitopes. None of the epitopes were found in the five remaining protein open reading frames (ORFs) encoded by the HIV-1 genome (VIF, TAT, REV, VPU/VPX, and VPR). This distribution reflects, in large part, the overall size as well as degree of conservation of the different HIV-1 ORFs. Furthermore, visual inspection of the relevant 3D structures reveals that these epitopes encompass residues that are important either for structural integrity and/or catalytic activity. For example, epitopes TLVDVGDAY and VIYQYMDDL from HIV-1 reverse transcriptase (POL) bear 6 and 4 residues, respectively, involved in substrate binding and catalysis [27]. The penalty for mutations at any of these sites vis-a-vis viral fitness presumably precludes ready development of escape variants.

**Table 2:** CTL epitopes conserved among HIV-1 proteomes

**EPITOPE**	**C-(1)**	**Source (2)**	**Position (3)**	**HLA I Restriction (4)**	**Predicted HLA I Restriction (5)**	**PPC(6)**
SPRTLNAWV	K	GAG:p24	148–156:16–24	B0702	B0702 B3501 B5101 B5102 B5103 B5301 B5401 B5502	0.35
AVFIHNFKR	K	POL:Integrase	894–902:179–187	A0301	A0301 A1101 A3101 A3301 A6601 A6801	0.35
TLFCASDAK	A	ENV:gp160	51–59	A0301	A0301 A1101 A3101 A3301 A6801	0.32
FPVRPQVPL	R	NEF	68–76	B3501	A2902 B0702 B3501 B5101 B5102 B5103 B5301 B5401	0.32
RAMASDFNL	P	POL:Integrase	735–743:20–28	A0201	A0201 B2709 C0304	0.31
TLNAWVKVI	E	GAG:p24	151–159:19–27	A0201	A0201 A0202 A0203 A0204 A0206	0.29
VIYQYMDDL	Y	POL: Reverse Transcriptase	334–342:179–187	A0201	A0201 A0205 A0207 A0214	0.28
LVGPTPVNI	I	POL:Protease	132–140:76–84	A0201	A0201 A0202 A0205 A0209 B1501 B1516	0.27
TVLDVGDAY	F	POL: Reverse Transcriptase	262–270:107–115	B3501	B1501 B3501 B5701 C0304	0.26
PLVKLWYQL	E	POL: Reverse Transcriptase	576–584:421–429	A0201	A0201 A0202 A0203	0.26
TLNFPISPI	E	POL: Protease	152–160:96–1004		A0201 A0207	0.23
NTPVFAIKK	K	POL: Reverse Transcriptase	212–220:57–65	A0301	A0301 A6601 C0102	0.22
EKEGKISKI	G	POL: Reverse Transcriptase	197–205:42–50	B5101	B2701 B3801 B39011 B3909 B4402 B5101 B8	0.19
LLWKGEGAV	V	POL: Reverse Transcriptase	956–964:241–249	A0201	A0201 A0204 A0205 A0209	0.18
LTFGWCFKL	V	NEF	137–145	A0201	A0201	0.18
YQYMDDLYV	G	POL: Reverse Transcriptase	336–344:181–189	A0201	A0201	0.18
GPKVKQWPL	T	POL: Reverse Transcriptase	173–181:18–26	B0801	B0702 B0801 B3501 B8	0.17
RAIEAQQHL	L	ENV:gp41	557–565:46–54	B5101 B1501 C0304	B1501 B1517 B5101 C0304	0.13
GLNKIVRMY	S	GAG:p24	269–277:137–145	B1501	A0203 A1 B1501	0.13
YFPDWQNYT	P	NEF	120–128	A1 B3701 B5701	A1 B3701 B5701	0.07
WYIKIFIMI	V	ENV:gp41	680–688:680–688	A2402	A0203 A0206 A2402	0.05
YVDRFFKTL	R	GAG:p24	296–304:164–172	A2601	A0203 A0204 A0207 A2601 B3801	0.05
FVNTPPLVK	L	POL: Reverse Transcriptase	571–579:416–424	A1101	A1101	0.05
KIQNFRVYY	R	POL:Integrase	934–942:219–227	A3002	A1 A3002	0.03
DRFFKTLRA	E	GAG:p24	298–306:166–174	B1402	B1402 B2701 B2702 B2703 B2704 B2705 B2709	0.03

1) Most proximal C-terminal flanking amino acid residue to the epitope

2) HIV-1 ORF

3) Position in mature protein.

4) HLA-1 restriction of the epitope as retrieved from the Los Alamos HIV database

5) Predicted HLA-1 binding profiles of epitopes were obtained using PSSMs [16].

6) Protection Population Coverage (PPC) was computed for 5 ethnic groups (Black, Caucasian, Hispanic, North American Natives and Asian) in the USA population. Each PPC value shown in the table is that of the ethnic group with the lowest PPC for that specific HLA I combination.

### A minority of HIV-1 patients responds to HIV-1 specific CTL peptide pools

That as few as 5 peptides may provide the requisite PPC largely stems from their ability to bind to many different HLA I molecules. The peptide SPRTLNAWV itself is anticipated to bind to eight distinct HLA I allelic variants, offering a PPC ranging from 35% in Asians to 52% in North American Indians. Such promiscuous MHCI-peptide binding often resides in previously defined HLA I supertypes (i.e. alleles with similar peptide binding specificity [28]), e.g., the peptide SPRTLNAWV binds to the B7 supertype and the peptide AVFIHNFKR to the A3 supertype. Nevertheless, binding promiscuity is not exclusively confined to HLA I supertypes. For instance, the peptide EKEGKISKI is predicted to bind to the alleles B2701, B3801 B39011, B3909, B4402, B5101 and B0801, which do not conform to any known supertype, and peptide LVGPTPVNI is predicted to bind to several alleles within the A2 supertype (A0201 A0202 A0205 A0209) but additionally to other non-related alleles of the HLA-B locus (B2701 and B3801).

**Figure 1:**
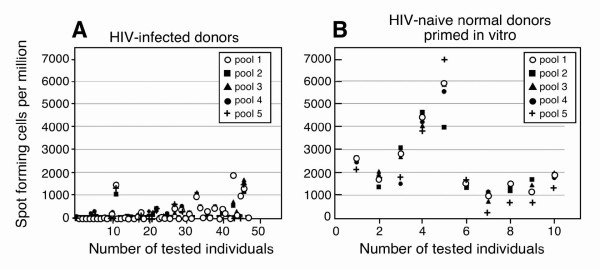
**Antigen-specific responses to conserved HIV-1 peptide pools in HIV-1-infected and naive individuals**. Conserved HIV-1 peptides were collected in five pools [1–5]. CTL responses of 47 immunocompetent HIV-1 infected patients to each peptide pool were analyzed by IFNγ ELISPOT (Panel A). The same HIV-1 peptide pools were used to prime PBMCs from naïve individuals and after two stimulations, similarly tested by IFNγ ELISPOT (Panel B).

In order to assess the accuracy of the predicted PPC, five peptide pools were created, each solubilized in DMSO and combining CTL epitopes (200 μM each) to provide a PPC ≥ 95 % (peptides included in the pools are shown in Table 3). Peptide 1 pool contained all 25 selected peptides whereas peptide pools #2 (15 peptides), #3 (13 peptides), #4 (11 peptides), #5 (7 peptides) comprising  55, 13, 11, 5, 2, respectively, of distinct 5-peptide combinations producing ≥ 95% PPC. Peptides pools 1–5 were designed so that peptides in the smaller pools are contained in the larger pools. CD8+ T cell responses to these pools were first checked by IFNγ ELISPOT assay using PBMC samples from a cohort of 47 HIV-1 infected patients. Patients were largely heterogeneous with regard to their HLA I background and included those diagnosed during acute or chronic HIV-1 with or without HAART therapy and some long-term non-progressors (Table 4). Furthermore, these patients were immunocompetent as judged by their ability to elicit responses to a pool of CMV, EBV and Influenza virus peptides and/or PHA [29]. A summary of the patient antigen-specific CD8 responses is given in Figure 1. Surprisingly, the percentage of patients that exhibit responses to the tested pools was far lower than expected, and ranged from 31% to 45%. There were no significant differences to HIV-1 peptide pools between HIV-1 infected patient groups (p < 0.0001) (see Figure 1A and Additional file 2).

**Table 3:** CTL peptides pools used in this study

**Pool**	**Peptides**	5-PC*	**Peptide Sequences**
#1	25	55	Those shown in Table 1
#2	15	13	FPVRPQVPL, KIQNFRVYY, AVFIHNFKR, GPKVKQWPL, LVGPTPVNI, TLNAWVKVI, GLNKIVRMY, TLFCASDAK, VIYQYMDDL, PLVKLWYQL, SPRTLNAWV, YVDRFFKTL, YFPDWQNYT, RAIEAQQHL, FVNTPPLVK
#3	13	11	FPVRPQVPL, KIQNFRVYY, AVFIHNFKR, GPKVKQWPL, LVGPTPVNI, TLNAWVKVI, GLNKIVRMY, TLFCASDAK, VIYQYMDDL, PLVKLWYQL, SPRTLNAWV, YVDRFFKTL, YFPDWQNYT
#4	11	5	FPVRPQVPL, KIQNFRVYY, AVFIHNFKR, GPKVKQWPL, LVGPTPVNI, TLNAWVKVI, GLNKIVRMY, TLFCASDAK, VIYQYMDDL, PLVKLWYQL, SPRTLNAWV
#5	7	2	FPVRPQVPL, KIQNFRVYY, AVFIHNFKR, GPKVKQWPL, LVGPTPVNI, TLNAWVKVI, GLNKIVRMY

*5-PC: distinct 5-peptide combinations in the peptide pool each providing ≥ 95% PPC

**Table 4:** HIV-1 patient information and CTL responses to peptide pools

	**PATIENT INFORMATION**							CTL RESPONSES TO PEPTIDE POOLS AS DETERMINED BY INTERFERON-GAMMA ELISPOT (SFC*)														
**PATIEND ID**	**HLA TYPE**	DISEASE STAGE*	HAART*	**ON HAART SINCE**	**INFECTED SINCE**	VL*	PBMC DATE*	**Pool 1**	**Pool 1**	**Pool 2**	**Pool 2**	**Pool 3**	**Pool 3**	**Pool 4**	**Pool 4**	**Pool 5**	**Pool 5**	CEF*	**NEG**	**NEG**	**NEG**	**POS**

1	Unavailable	Unavailable	Unavailable	NA	Unavailable	Unavailable	6/22/04	1860	1610	530	510	700	620	10	10	10	10	0	0	0	0	PPP
2	Unavailable	Unavailable	Unavailable	NA	Unavailable	Unavailable	2/17/04	910	960	20	0	40	40	0	0	10	10	0	20	0	0	PPP
3	Unavailable	Chronic	No	NA	Unknown	>750000	2/19/04	0	0	0	10	0	0	0	0	0	0	675	0	0	0	PP
4	Unavailable	Chronic	No	NA	Unknown	>750000	2/26/04	0	0	0	0	0	0	0	0	0	0	70	0	0	0	PPP
5	Unavailable	Chronic	No	NA	Unknown	12905	3/4/04	110	150	60	80	90	70	50	30	30	40	266	60	40	50	PPP
6	Unavailable	Chronic	Yes	4/18/01	Unknown	176	2/26/04	180	130	150	200	150	150	210	250	100	120	163	0	0	0	PPP
7	Unavailable	Chronic	Yes	1/26/03	Unknown	6030	2/19/04	0	0	0	0	0	0	0	0	0	10	60	0	0	0	PPP
8	Unavailable	Chronic	Yes	NA	Unknown	>750000	2/24/04	0	0	0	0	0	0	0	0	0	0	>1000	0	0	0	PPP
9	Unavailable	Chronic	No	NA	Unknown	>500000	2/19/04	0	0	10	0	30	20	170	150	0	0	540	0	0	0	PP
10	Unavailable	Chronic	No	NA	Unknown	35100	2/26/04	1440	1010	1010	760	1040	1070	1050	1080	1270	1160	>1000	10	20	40	PPP
11	Unavailable	Chronic	No	NA	Unknown	236000	3/2/04	10	10	30	30	20	20	30	30	30	40	20	10	30	10	PPP
12	Unavailable	Chronic	No	NA	Unknown	9030	2/19/04	0	0	0	0	0	0	0	0	0	0	140	0	0	0	PP
13	Unavailable	Chronic	No	NA	Unknown	12000	3/4/04	260	210	190	160	180	210	150	210	150	160	>1000	10	20	0	PPP
14	A11/74, B35/44, C4/5	Chronic	No	NA	Unknown	74	4/20/04	0	0	0	0	0	0	0	0	0	0	833	0	0	0	PPP
15	A30/33, B13/14, C5/8	Chronic	No	NA	Unknown	122	4/20/04	60	90	20	0	20	10	20	10	20	20	0	0	10	10	PPP
16	A25/32, B18/40, C2/12	Chronic	No	NA	Unknown	12700	4/20/04	420	410	340	350	220	370	550	490	620	500	0	10	10	0	PPP
17	a2/30, b44/57, c5/18	Chronic	No	NA	Unknown	49	4/27/04	380	380	420	470	330	410	490	500	360	250	953	0	0	0	PPP
18	A1/74, B57/81, C7/18	Chronic	No	NA	Unknown	845	6/3/04	0	20	0	20	10	10	10	0	20	20 ND	0	0		10	PP
19	A3/33, B15/49, C7/14	Chronic	No	NA	Unknown	49	6/16/04	350	350	170	140	230	270	220	240	110	90 ND	0	0		10	PPP
20	A29, B44, C16	Chronic	No	NA	Unknown	807	6/16/04	210	180	130	180	200	260	0	20	0	0 ND	0	10		0	PPP
21	A1/24, B13/57, C6	Acute	No	NA	Unknown	5490	6/22/04	130	120	110	120	270	230	0	0	0	0	60	0	10	0	PPP
22	A1/24, B8/35, C4/7	Acute	Yes	7/21/03	2002, May	<50	2/17/04	130	100	30	50	70	40	120	100	70	50	347	0	0	0	PPP
23	A24/32, B35/41, C4/17	Acute	Yes	6/1/02	2002, Jun	<50	2/19/04	0	0	0	0	0	0	260	300	200	140	146	0	0	10	PPP
24	A2/31, B40/44, C3/16	Acute	Yes	9/2/03	2003, Aug	<400	2/26/04	0	0	0	0	0	0	0	0	0	0	440	0	0	0	PPP
25	A11/30, B15/35, C3/4	Acute	Yes	12/30/03	2003, Dec	<400	2/26/04	20	30	10	30	20	30	30	40	10	30	396	20	0	20	PPP
26	A11/24, B35/38, C4/7	Acute	Yes	5/19/99	1999, may	<50	3/1/04	0	0	0	0	0	0	0	0	0	0	60	0	20	0	PPP
27	A1/24, B14/35, C4/8	Acute	Yes	9/8/03	2003, JUL	<400	3/1/04	40	50	40	30	30	60	10	50	30	10	100	0	0	20	PPP
28	A1/11, B18/52, C12	Acute	Yes	8/25/99	1999, SEP	<50	3/9/04	0	0	0	0	0	0	0	0	0	0	520	0	0	0	PPP
29	A3/68, B14/44, B8/16	Acute	Yes	9/2/03	2003, AUG	<50	3/9/04	250	320	50	50	40	20	0	0	20	20	140	0	0	0	PPP
30	A1/32, B7/44, C7/16	Acute	Yes	2/18/04	2004, FEB	5,440	4/20/04	40	20	100	90	120	110	170	120	180	220	433	0	0	0	PPP
31	A2/3, B7/44, C5/7	Acute	Yes	8/1/97	1997-AUG	149	4/20/04	20	0	0	0	0	0	10	0	0	0	443	0	10	0	PP
32	A33, B53, C4	Acute	Yes	12/14/00	2000-NOV	<50	4/22/04	0	0	0	0	0	0	0	0	0	0	0	0	0	0	PPP
33	A1/68, B7/8, C7/7	Acute	Yes	4/23/04	2004-APR	853	5/6/04	10	10	0	0	0	0	0	10	0	0	ND	0	0	0	PPP
34	A3/24, B40/57, C3/7	Acute	Yes	11/5/03	2003-NOV	<50	5/12/04	220	170	190	170	190	120	10	30	0	10	0	30	10	20	PPP
35	A26/68, B44/71, C3/7	Acute	Yes	12/22/98	1996-NOV	<50	5/13/04	0	0	0	20	10	10	0	10	10	10	20	10	10	10	PPP
36	A2/26, B53/57, C4/18	Acute	Yes	5/24/01	2001, MAY	<400	6/23/04	0	0	0	0	0	10	0	0	0	0	0	0	10	0	PPP
37	A2/74, B51/53, C4/14	Acute	Yes	8/22/01	2001, AUG	<50	6/22/04	0	10	0	10	20	20	0	20	0	10	986	0	0	0	PPP
38	A1/2, B8/15, C3/7	Acute	Yes	3/9/99	1999, MAR	1,111	5/19/04	420	430	480	420	500	410	430	410	430	480	326	0	0	0	PPP
39	A1/2, B7/8, C7/7	Acute	No	7/22/02	2002, JUL	<400	3/2/04	10	20	10	10	0	10	10	0	0	10	>1000	0	0	10	PPP
40	A23/24, B44/60, C3/14	Acute	No	NA	2004, FEB	1,580	3/15/04	210	220	90	90	80	100	0	0	0	0	70	0	20	0	PPP
41	A1/3, B8/15, C7	Acute	No	NA	2003, AUG	56,100	4/14/04	260	210	380	480	360	290	300	430	430	440	ND	0	0	0	PPP
42	A2/31, B15/51, C3/15	Acute	No	NA	2003-MAY	2,620	6/3/04	410	430	480	410	490	430	440	410	400	470	116	0	0	20	PPP
43	A3/24, B14/35, C8/12	Acute	No	NA	2000-MAY	883	5/26/04	250	250	40	30	20	40	40	40	40	30	80	0	0	10	PPP
44	A1/3, B35/40, C4/15	Acute	No	NA	2003-OCT	121,000	5/13/04	910	1150	1010	1010	1100	1080	980	1330	930	1090	260	0	0	0	PPP
45	A3/41, B14/35, C8/12	Acute	No	NA	2002, FEB	29,000	6/22/04	950	1090	30	40	40	60	40	40	50	60	143	0	0	0	PPP
46	A2/32, B15/39, C4/15	Acute	No	NA	2001, OCT	48,000	6/23/04	1200	1200	1150	1460	1660	1300	1460	1290	1390	1540	160	0	10	10	PPP
47	A3/68, B35/44, C4/7	Acute	No	NA	1999, AUG	667	5/26/04	10	30	0	30	10	20	0	20	10	0	ND	0	20	10	PPP

CTL responses were determined by the interferon-gamma elispot assay

SFC*: Spot Forming Colonies

DISEASE STAGE*: refers to the stage at the time of diagnosis

HAART*: Patients were receiving HAART treatment at the time of PBMC collection

VL*: Viral Load at the time of PBMC sampling in copies/ml

PBMC DATE*: Date PBMC samples were collected

CEF: Response to CMV, EBV and Influenza A peptide pools.

POS is a PHA response where PP = high and PPP = highest responses related to non-stimulated controls. Both PP and PPP are equivalent to responses form normal individuals.

NA, Not Applicable

### T cell responses obtained from all HIV-1 naive donors primed with HIV-1 specific CTL peptide pools

Errors in the prediction of HLA I binding profiles of epitopes may have contributed to the above discrepancies between expected and experimental responses. Nonetheless, prediction of peptide-MHCI binding *per se *is quite accurate, and in general experimental HLA I binding can be confirmed for at least 75% of HLA I predicted binders [30]. Thus, while we do not discard the possibility that errors in predicting epitopes by HLA I binding profiles might exist, they cannot account for the great divergence between the expected CTL responses and those observed experimentally. More likely, functional impairment of CTL in chronically HIV-1-infected patients may render CTLs unable to produce IFNγ [31,32]. In addition, the specificities of CTL responses change during disease progression [33]. Those targets of CTL responses observed in individuals during the course of any viral infection are determined by subtle immunodominance patterns influenced by many factors including other HLA molecules and previous pathogenic encounters [34]. To address these possibilities, we analyzed responses in HIV-1 naive individuals by priming PBMC of ten donors at high cell density with HIV-1 peptide-pool pulsed autologous Langerhans-like cells followed by restimulation with peptide pulsed fresh PBMCs. We tested the resulting HIV-1 peptide pool specific T cell lines by ELISPOT analysis. (Figure 1B) All donors generated IFNγ with a magnitude significantly greater (p < 0.0001) than ELISPOT responses generated by HIV-1-infected subjects.

### Characterization of A0201 restricted responses in HIV-1 naive individuals

**Figure 2:**
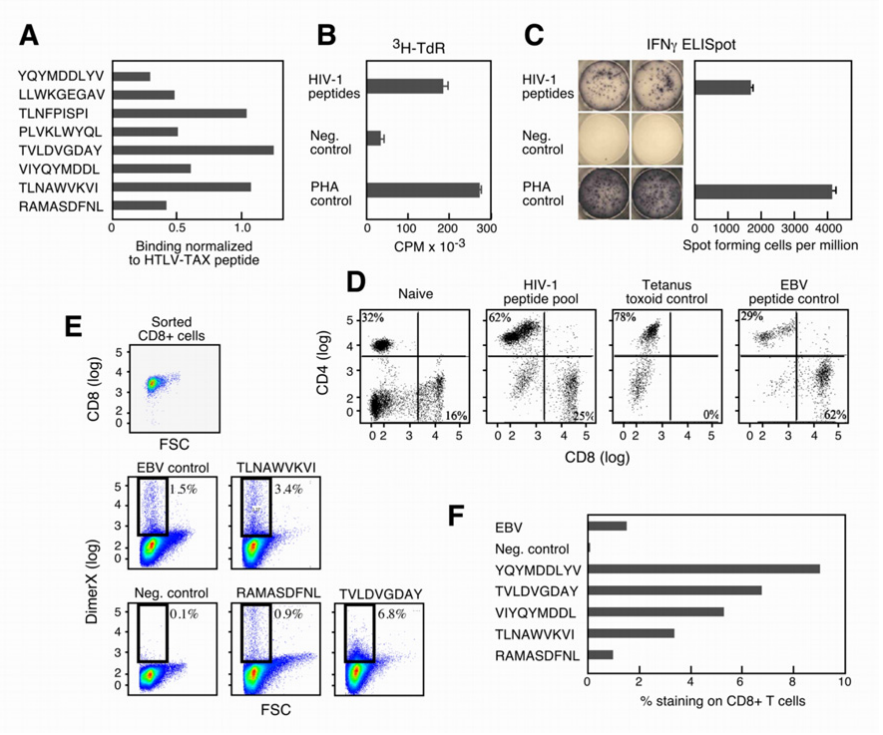
**Generation and characterization of HLA-A0201 restricted HIV-1 peptide-specific T cell lines from uninfected individuals**. Binding of HIV-1 peptides to A0201 was tested using the T2 cell line (panel A). These HIV-1 peptide-stimulated T cells generated antigen-specific proliferative responses (panel B). ELISPOT responses to HIV-1 peptide-loaded donor PBMCs or PHA stimulation (positive control) as well as to non-peptide loaded donor PBMCs (negative control) (Panel C). Flow cytometric analysis of CD4 and CD8 T cells in HIV-1 peptide-specific T cell lines and indicated control T cell lines (Panel D). CD8^+ ^T cells were sorted from the HIV-1 specific T cell line after multiple stimulations *in vitro *and their peptide specificities determined by individual peptide-loaded DimerX staining (Panel E). In Panel E, the negative control straining was obtained using the HIV-1 specific T cell line and a non-relevant peptide loaded DimerX. The EBV positive control was obtained using donor PBMC's stimulated with 1uM BMLF-1 EBV peptide for one week and then stained with EBV peptide loaded Dimer X. One to nine percent of CD8 T cells were specific for each one of the selected HIV-1B peptides (Panel F).

To further evaluate specific T cell responses, we selected the most common HLA subtype, A0201 for detailed analysis. Eight HIV-1 peptides with A0201 restriction (Table 2) were pulsed individually onto the TAP deficient A0201+ T2 hybridoma cell line to analyze functional peptide binding. A positive control peptide derived from the HTLV-TAX protein [(amino acids 11–19. LLFGYPVYV)] was used to normalize positive peptide binding (value = 1). A predicted A0301 restricted HIV-1 peptide was used as a negative control (value = 0). Each HIV-1 peptide was found to bind A0201 as shown by increased A0201 surface expression on peptide-pulsed T2 cells (Figure 2A). Subsequently, A0201 restricted HIV-1 peptides were combined in a pool and were used to prime an A0201 donor, resulting in selective proliferation to the HIV-1 peptide pool compared to control (Figure 2B). These cells produced IFNγ upon stimulation with HIV-1 peptide-pulsed antigen-presenting cells (PBMCs) in ELISPOT assays (Figure 2C). After three rounds of stimulation, 25% of the cells in culture were CD8^+ ^and 62% were CD4^+ ^(Figure 2D). Given that ~20–30% of MHCI binding peptides are predicted to bind to MHCII as well, this result is not unexpected (data not shown). We also generated two control cell lines: the first, a tetanus toxoid specific T cell line with 78% CD4^+ ^and no CD8^+ ^T cells elicited by whole protein stimulation; and the second, an EBV specific T cell line with 62% CD8^+ ^and 29% CD4^+ ^cells elicited by stimulation with an immunodominant EBV epitope BMLF1 (GLCTLVAML). CD8^+ ^T cells were sorted from the HIV-1 specific T cell line after several stimulations *in vitro *(Fig. 2E, first plot) and their peptide specificities determined by individual peptide-loaded DimerX staining (Figure 2E). One to nine percent of CD8^+ ^T cells were specific for each one of the selected HIV-1B peptides (Figure 2F).

### Cytotoxic activity of HIV-1 peptide-specific CD8+ T cells derived from HIV-1 naive donors

**Figure 3:**
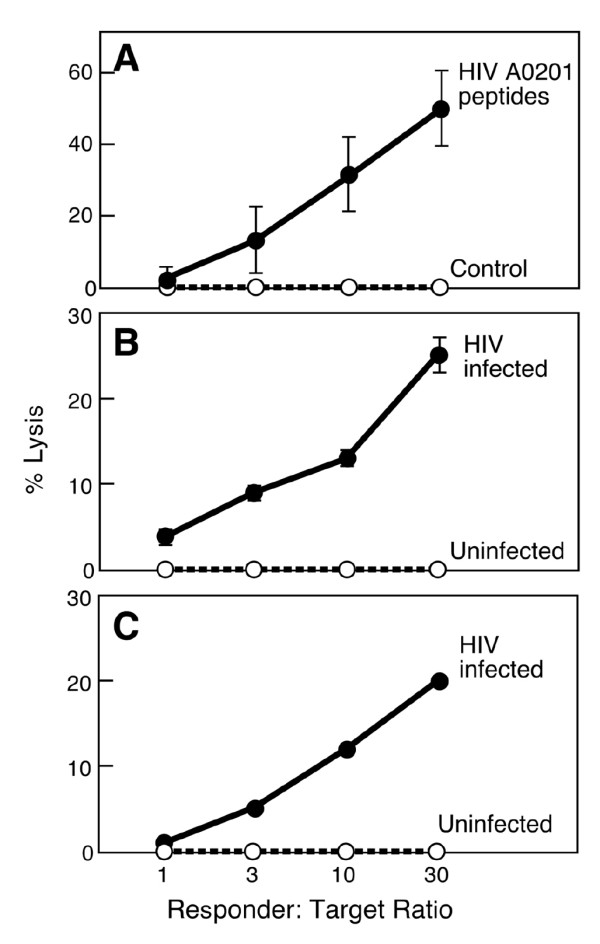
**Cytotoxic activity of HIV-1 peptide-specific A0201 restricted T cells from normal donors**. CTL were generated against a pool of eight A0201 restricted HIV-1 peptides using PBMC. HIV-1 specific CTL lysed HIV-1 peptide loaded T2 cells (solid symbols) but not the irrelevant peptide loaded T2 cells (open symbols) in ^51^Cr release assay (Panel A). HIV-1 specific CTL generated from 2 different naïve donors lysed HIV-IIIB- infected T1 cells (solid symbols) but not uninfected T1 cells (open symbols) 2 and 3 days after acute infection, in a ^51^Cr release assay (Panels B and C, respectively).

**Figure 4:**
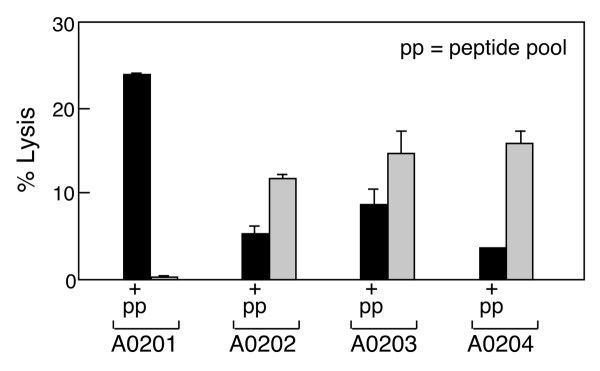
**Allele-restricted fine specificity of CTL directed at HIV-1 peptides**. HIV-1 peptide-specific T cells were generated from an A0201 donor using predicted A0201-restricted HIV-1 peptides (Table 1 and Fig. 1). HIV-1 peptide-specific T cells killed the A0201 transfected 721–221 cell line in a peptide-specific manner by ^51^Cr release assay. To test possible cross-reactivity of A0201 restricted HIV-1 peptide-specific CTL to other A02 alleles, we utilized A0202, A0203 and A0204 transfected 721–221 cells as targets. A0201 restricted HIV-1 peptide specific CTLs killed HIV-1 peptide loaded A0201 transfected 721–221 targets at a 10:1 ratio.  +pp indicates addition of peptide pool, with solid bar = lysis upon +pp and gray bar = lysis with no peptide pool addition.

Next, to test for potential cytolytic activity of the HIV-1 peptide-specific T cell lines, T2 hybridoma cells were pulsed with A0201 restricted HIV-1B peptide pools and specific lysis was determined by standard ^51^Cr release assay. As shown in Figure 3A, the HIV-1 peptide-specific CD8^+ ^T cells killed A0201 restricted HIV-1 peptide pool-pulsed T2 cells with ~50% specific lysis at an E:T ratio of 30:1. The same T2 cells loaded with HTLV-TAX peptide as a negative control were not lysed. To determine whether HIV-1 peptide-specific A0201 restricted CTL can recognize and lyse HIV-1 -infected cells, we infected the T1 hybridoma line (A0201+, B5101^+^, CD4^+^) with HIV-IIIB virus and then determined whether HIV-1 peptide specific A0201 restricted CTL can recognize HIV-IIIB infected T1 cells and kill target cells in a ^51^Cr release assay. HIV-1 specific CD8 T cells from two different A0201 donors recognized and killed 20–28% of HIV-1 infected T1 cells 2 and 3 days after infection at an E:T ratio of 30:1 while uninfected T1 cells were not lysed (Figures 3B and 3C). We also noted that although some of these peptides (for example, TLNAWYKVI) bind to related alleles, CD8^+ ^T cells stimulated from the A0201* donor fail to specifically lyse peptide-pulsed A0202, A0203 or A0204 targets (Figure 4). Thus, a given CTL response is less degenerate than peptide binding to MHC.

As shown in Figure 4, A0201 restricted HIV-1-specific CTL demonstrate alloreactivity and kill A0202, A0203 and A0204 transfected 721–221 targets in the absence of HIV-1 peptide loading. Moreover, in the presence of HIV-1 peptides, alloreactivity against A0202, A0203 and A0204 transfected 721–221 targets decrease as shown by diminished lysis. These results suggest that A0201 restricted HIV-1 peptides bind to A0202, A0203 and A0204, replacing possible endogenous peptides that mediate much of the evident alloreactivity. The analysis of the A0204 transfectant is a clear example of this phenomenon.

## Conclusion

The present report supports the notion that T cell responses in HIV patients are impaired and argues for optimizing the composition of epitope-based vaccines using data obtained from naive individuals. However, the responses present in HIV-1-infected subjects and the ability to grow peptide-specific CD8 T cells from naive individuals are not directly comparable. Further experimental work shall include analysis of the T cell responses in naive and HIV-1-infected patients using in vitro stimulated CD8 T cells and HIV-specific CTL epitopes. Perhaps this approach will elucidate whether clonal deletion or anergy exists, for example.

Overall, these findings demonstrate that normal, HIV-1 naive individuals possess T cells capable of recognizing and expanding against conserved segments of the HIV-1 proteome. Although only a minority of chronically HIV-1-infected patients responds to pools of such epitopes, viral protective responses can be elicited from all normal subjects examined. Thus, the absent response in HIV-1-infected patients is not a consequence of intrinsic holes within the human T cell repertoire, but rather the sequelae of chronic exposure to antigens, immunodominance patterns and/or cellular dysfunction. In this regard, the ability of HIV-1 to drive cellular responses away from relevant, invariant segments capable of affording protection toward mutable epitopes that escape CTL-based destruction is reminiscent of the anti-HIV-1 antibody response in chronically infected patients [4]. In the latter, antibodies are directed to the variable loops of the HIV-1-gp160 envelope protein rather than invariant potential neutralization sites. Thus, viral escape mechanisms send both T cells and B cells off in unproductive directions. That human T cell responses can be elicited from virus-naive individuals to conserved segments of the HIV-1 proteome argues that CTL-directed vaccines may target these epitopes, and underscores the importance of assessing immune responses of normal individuals as well as HIV-1-infected patients. Beyond HIV-1, immunoprotection against other chronic infectious diseases such as malaria as well as neoplastic disorders may benefit from a paradigm shift in vaccine design, focusing on potential immune responses in normal rather than in patient populations.

## List of abbreviations used

AIDS, acquired immunodeficiency syndrome; CPF, combined phenotypic frequency; CTL, cytotoxic T lymphocytes; HAART highly active antiretroviral therapy; HIV-1, human immunodeficiency virus-1; PPC, population protection coverage; SIV, simian immunodeficiency virus

## Competing interests

The author(s) declare that they have no competing interests.

## Authors' contributions

PAR: Contributed to the design of the study, carried out all bioinformatic analysis and produced the manuscript

DBK: Contributed to the experimental design of the work, carried out all immunology experiments and helped with paper writing.

REH: Established the single HLA I allele 721221 transfectant cell lines used in this study with the help of PAR.

PA: Assisted with live HIV infection studies

DG: Contributed to the design of HIV infected/autologous cell-CTL analyses

ELR: Designed and oversaw the study and contributed to the manuscript.

## Supplementary Material

Additional File 1 HIV-1 consensus proteome where variable residues are masked with the "." symbol Click here for file

Additional File 2 Statistics on peptide responses in ELISPOT assay for the three subgroups Click here for file
