# Supplementary material for: Elicitation from virus-naive individuals of cytotoxic T lymphocytes directed against conserved HIV-1 epitopes
Source: Med Immunol. 2006 May 18;5:1. doi: 10.1186/1476-9433-5-1 (PMC1559620; doi:10.1186/1476-9433-5-1)
Supplement: Additional File 1 — HIV-1 consensus proteome where variable residues are masked with the "." symbol [file 1476-9433-5-1-S1.pdf]

Figure 1S. HIV-1 consensus proteome where variable residues are masked with the "." symbol

```
>ENV

MRV.....N.....W.WG..........C.......LWVTVYYGVPVW..A.TTLFCASDAKAY..E.HNVWATHACVPTDPNPQE

..L.NV.FNMWKN.MV.QMH.DIISLWDQSLKPCVKLTPLCVTL..........................................

NCSFN.TT...DK.....ALFY..D.V................YRLI.CNTS.ITQACPKV.F.PIPIHYCAPAGFAILKC....F

NGTG.C.NVSTVQCTHGI.PVVSTQLLLNGSLA......IRS.N.T.N.K.IIV.L...V.I.C.RP..N............PG..

.Y......G.IR.A.C......W...L......L..........I.F.....GGD.EI..H.FNC.GEFFYCNT..LFN.......

...........................I.L.CRIKQIIN.WQ.VG.AMYAPPI.G.I.C.SNITGLLLTRDGG.............

..FRP.GGDMRDNWRSELYKYKVV.I.PLG.APT.AKRRVV.RE..AVG..GA.FLGFLGAAGSTMGAAS.TLTVQAR.LLSGIVQ

QQ.NLLRAIEAQQHLL.LTVWGIKQLQARVLA.ERYL.DQQLLGIWGCSGK.ICTT.VPWN.SWS.......IW.NMTW..W..EI

.NYT..IY.LIE.SQ.QQE.NE..LL.LD.W..LW.WF.IT.WLWYIKIFIMIVGGLIGLRI.F.VLS.VNRVRQGYSPLSFQT..

P.....DRP..IEE.GGE.DR.RS.RLV.GFL.L.W.DLR.LCLFSYH.LRD..LI..R.VE.LG.........GWE.LKY...L.

.YW..ELK.SA..L....AIAVA..TDR.IE..QR..RA...IP.RIRQG.E.AL.

>TAT

MEPVDP.LEPW.HPGSQP.T.C..CYCK.CC.HC..CF..KGLGISYGRKKR..RR..P.....HQ....KQP.........TG..

E.KK.V...........

>VPR

MEQAPEDQGPQREP.NEW.LELLEELK.EAVRHFPR.WLH.LGQ.IY.TYGDTW.GVEAIIR.LQQLLF.HFRIGC.HSRIGI...

.RR.RNG..RS

>REV

MAGRSG..DE.LL...R.IK.LYQSNP.P...GTRQARRNRRRRWR.RQ.QI..IS.RIL...LGR..EPVPLQLPP.ERL.L...

E..G..GT.................E....L......

>VPU/VPX

M............L.VA..I..I.VW.I..IE......Q.KI..L..RI.ERAEDSGNES.GD.EEL......G...........

>NEF

MGGKWSK....GW...RERM.............AA.GVGA.S.DL...GA.TSSNT...NA.CAWLEAQE...E.VGFPVRPQVPL

RPMTYK.A.DLSHFLKEKGGLEGLI.S..RQDILDLWVYHTQGYFPDWQNYTPGPG.R.PLTFGWCFKLVPV.P..VEE

AN.GENN.LLHP...HGM.D.E.EVL.W.FDS.LA..H.ARE.HPEYYK.C

>POL

FFRE.LAF.QGEAR.F..EQ.............R.L...............E.G.....G.......FPQITLWQRPLV..KIGGQ

..EALLDTGADDTVLE...LPG.WKPKMIGGIGGFIKVRQYDQI.IEICG.KAIGTVLVGPTPVNIIGRN.LTQIGCTLNFPISPI

ETVPVKLKPGMDGPKVKQWPLTEEKIKAL..IC.EMEKEGKISKIGPENPYNTPVFAIKKKDSTKWRKLVDFRELNKRTQDFWEVQ
```

LGIPHPAGLKKKKSVTVLDVGDAYFSVPLD..FRKYTAFTIPS.NNETPGIRYQYNVLPQGWKGSPAIFQ.SMTKILEPFR..NP.
.VIYQYMDDLYVGSDLEIGQHR.KIEELR.HLL.WGFTTPDKKHQKEPPFLWMGYELHPDKWTVQPI.LP.K.SWTVNDIQKLVGK
LNWASQIY.GIKVKQLCKLLRG.KALTD.V.LT.EAELELAENREILK.PVHGVYYDP.KDL.AE.QKQG..QWTYQIYQEP.KNL
KTGKYA..R.AHTNDVKQLTE.VQKI..ESIVIWGK.PKF.LPIQKETWE.WW.EYWQATWIPEWEFVNTPPLVKLWYQLE.EPI.
GAETFYVDGAANRETK.GKAGYVTDRGRQK.V.L..TTNQKTEL.AI.LALQDSG.EVNIVTDSQYALGIIQAQPD.SESE.V.QI
IE.LI.KE.VYL.WVPAHKGIGGNEQVDKLVS.GIRKVLFLDGIDKAQEEHE.YH.NWRAMASDFNLPPIVAKEIVASCDKCQLKG
EAMHGQVDCSPGIWQLDCTHLEGK.ILVAVHVASGYIEAEVIPAETGQETAYF.LKLAGRWPVK.IHTDNG.NFTS..VKAACWWA
.I.QEFGIPYNPQSQGVVESMNKELKKIIGQVRDQAEHLKTAVQMAVFIHNFKRKGGIGGYSAGERIIDIIATDIQTKELQKQI.K
IQNFRVYYRDSRDP.WKGPAKLLWKGEGAVVIQDNSDIKVVPRRKAKIIRDYGKQMAG.DCVA.RQDED

>VIF

MENRWQVMIVWQVDRMRI.TW.SLVKHHMY.S.KA..W.YRHHYES.HPR.SSEVHIPLG.A.LV..TYWGL.TGER.WHLG.GVS
IEWR...Y.TQVDP.LADQLIH..YFDCF..SAIR.AILG..V.P.C.Y.AGHNKVGSLQYLAL.AL..P...KPPLPS
V.KL.EDRWNKPQKT.G.RG.HTMNGH

>GAG

MGARASVL.GG.LD.WEKIRLRPGGKK.Y..KHLVWASRELERFA.NP.LLET.EGC.QI..QLQP.L.TGSEEL.SL.NTVATLY
CVH..I.V.DTKEAL.K.EE.Q.....K...................SQNYPIVQN.QGQMVHQ..SPRTLNAWVKVIEEKAFSPE
VIPMF.ALSEGATPQDLN.MLN.VGGHQAAMQMLKDTINEEAAEWDR.HP..AGP..PGQMREPRGSDIAGTTSTLQEQI.WMT.N
PPIPVG.IYKRWIILGLNKIVRMYSP.SILDIRQGPKEPFRDYVDRFFKTLRAEQATQ.VKNWMTETLLVQNANPDCK.ILKALG.
GATLEEMMTACQGVGGP.HKARVLAEAMSQ........IMMQR.NF.......KCFNCGKEGH.ARNCRAPRKKGCWKCG.EGHQM
KDCTERQANFLGKIWPS.KGRPGNF.Q.R..PTAPP.E.....EE........KQ...........PL.SLKSLFG.DP...
