# Supplementary material for: Elicitation from virus-naive individuals of cytotoxic T lymphocytes directed against conserved HIV-1 epitopes
Source: Med Immunol. 2006 May 18;5:1. doi: 10.1186/1476-9433-5-1 (PMC1559620; doi:10.1186/1476-9433-5-1)
Supplement: Additional File 2 — Statistics on peptide responses in ELISPOT assay for the three subgroups [file 1476-9433-5-1-S2.pdf]

Table 1S. Statistics on peptide responses in ELISPOT assay for the three subgroups of HIV-1-infected subjects vs. controls

	a_inf				c_inf.				normal				no HAART						
	N	Min	Median	Max	N	Min	Median	Max	N	Min	Median	Max	N	Min	Median	Max	p1	p2	p3
pool1	26	0.0	20.8	1193.3	11	0.0	0.0	1201.7	10	933.3	1763.3	5546.7	8	0.0	156.7	408.3	0.0001	0.0004	<0.0001
pool2	26	0.0	17.5	1298.3	11	0.0	5.0	861.7	10	1040.0	1743.3	4346.7	8	0.0	131.7	445.0	0.0001	0.0004	<0.0001
pool3	26	0.0	23.3	1473.3	11	0.0	3.3	1031.7	10	693.3	1950.0	5560.0	8	0.0	236.7	370.0	0.0001	0.0004	<0.0001
pool4	26	0.0	7.5	1368.3	11	0.0	0.0	1041.7	10	1093.3	1590.0	5240.0	8	0.0	7.5	513.3	0.0001	0.0004	<0.0001
pool5	26	0.0	5.8	1458.3	11	0.0	0.0	1191.7	10	226.7	1673.3	7826.7	8	0.0	15.0	553.3	0.0002	0.0008	<0.0001

p1: c_infection vs. normal, p2: no HAART vs. normal, p3: a_infection vs. normal where a=subjects identified during acute infection, c = subjects with advanced chronic disease, and subjects "no HAART" are controlling viremia without therapy

note 1: Wilcoxon-Rank-Sum test is used for p-values

note 2: to take multiple comparisons into account, the significance level is set to 0.0167 (not 0.05). All comparisons are significant at the 0.0167 level.
